# Curcumin inhibits adverse psychological stress‐induced proliferation and invasion of glioma cells via down‐regulating the ERK/MAPK pathway

**DOI:** 10.1111/jcmm.16749

**Published:** 2021-06-25

**Authors:** Ping Wang, Xinwei Hao, Xiaohan Li, Yizhi Yan, Wenxiu Tian, Lin Xiao, Zhenming Wang, Junhong Dong

**Affiliations:** ^1^ Department of Molecular Biology and Biochemistry Weifang Medical University Weifang China; ^2^ Department of Clinical Laboratory Weifang People's Hospital Weifang China

**Keywords:** adverse psychological stress, curcumin, ERK1/2, glioma, norepinephrine

## Abstract

Curcumin is a natural polyphenol extracted from the rhizome of *Curcuma* that has an important antitumour effect, but its effect on adverse psychological stress‐induced tumour proliferation and invasion has not been reported to date. Here, we found that curcumin not only inhibited the growth of xenografts in chronically stressed nude mice, but also decreased the expression of matrix metalloproteinase (MMP)‐2/9 and CD147 in tumour tissues. Exogenous norepinephrine (NE) was used to stimulate glioma cells to simulate the stress environment in *vitro*, and it was found that curcumin inhibited the NE‐induced proliferation and invasion of glioma cells in a dose‐dependent manner. Further research found that the effects of NE on glioma cells could lead to the activation of the mitogen‐activated protein kinase (MAPK) signalling pathway through β‐adrenergic receptor, while curcumin suppressed the level of extracellular signal–regulated kinase (ERK)1/2 phosphorylation. In addition, blocking ERK1/2 expression with U0126 resulted in the down‐regulated expression of CD147, which further led to the decreased expression of MMP‐2 and MMP‐9. Curcumin could also inhibit the expression of cyclin D1/CDK4/6 and anti‐apoptotic protein Bcl‐2/Bcl‐XL induced by NE, and induced cell cycle changes and increased apoptosis. Therefore, curcumin may be a potential candidate drug for preventing and treating the progression of glioma induced by adverse psychological stress.

## 1 INTRODUCTION

A large number of clinical and epidemiological studies have shown that psychological stress is closely associated with the occurrence and progression of cancer. ^1^, ^2^, ^3^ Psychological stress promotes the production of catecholamines, such as norepinephrine (NE) and epinephrine (EPI), by activating the sympathetic nervous system (SNS). These stress hormones could promote the growth and invasion of various tumours, including ovarian, pancreatic and breast cancer. ^4^, ^5^, ^6^ Our previous studies also demonstrated that adverse psychological stress could promote the proliferation and invasion of glioma.^7^ Glioblastoma multiforme (GBM) is the most common malignant brain tumour in adults, with a survival rate of <5% in 5 years.^8^ At present, the standard treatments for glioma include surgical resection and radiotherapy. However, the invasive growth of GBM often leads to the inability to completely eradicate GBM, and adverse psychological stress promotes further deterioration.^9^ Therefore, further comprehensive and effective interventions are urgently needed to improve the treatment of patients with glioma.

Curcumin is a polyphenolic compound found in the rhizome of turmeric plants, which has various biological effects, including anti‐inflammatory, anti‐oxidation and anti‐infection effects.^10^, ^11^ In recent years, curcumin has been found to play a key role in inhibiting the initiation, progression and metastasis of several tumours,^12^, ^13^, ^14^ but its effect on stress‐induced tumour proliferation and invasion has not been reported to date. More importantly, curcumin is also the main ingredient of the Traditional Chinese Medicine formula Jieyu Pill, which is typically used for its mood stabilizing/enhancing properties, and therefore is mainly employed for the treatment of depression.^15^, ^16^ A recent study found that curcumin could enhance resistance to adverse stress and relieve stress‐induced anxiety‐like behaviour in mice.^17^ Thus, it was speculated that curcumin may play an important role in alleviating glioma progression, which is exacerbated by adverse psychological stress.

Several studies have shown that NE could induce the production of matrix metalloproteinases (MMPs) in tumour cells by acting on the adrenergic receptors on the surface of target cells. The gelatin‐degraded MMPs, including MMP‐2 and MMP‐9, are known to play an important role in the invasion and metastasis of cancer cells by degrading type IV collagen, which is the main component of the basement membrane.^18^ The activity of MMPs is regulated by a variety of mechanisms, including growth factors, cytokines and CD147. Accumulating evidence has revealed the role of CD147 in the development and progression of various cancer types, including glioma, ovarian cancer, renal cell carcinoma, laryngeal squamous cell carcinoma and skin cancer.^19^, ^20^, ^21^ As an MMP inducer, CD147 can also stimulate surrounding fibroblasts to secrete abundant MMPs to further enhance tumour cell invasion. Therefore, inhibiting the expression of MMPs and/or their upstream regulatory pathways may be critical in the treatment of malignant tumours such as glioma. Curcumin inhibits the expression of MMPs by targeting a variety of intracellular signalling pathways, including MAPK, nuclear factor‐κB (NF‐κB) and PI3K/Akt.^12^, ^13^, ^14^ MAPK is a crucial kinase that promotes proliferation and transmits stress signals. Its family members include ERK1/2, c‐Jun NH‐2 terminal kinase (JNK) and p38 MAPK. The MAPK signalling pathway is activated by the stress hormone NE in various tumours.^22^, ^23^ Our previous study found that the stress hormone NE could stimulate the progression of glioma cells by enhancing the activity of ERK and the expression of phosphorylated (p)‐ERK1/2,^7^ but it is unclear whether curcumin has an inhibitory effect on this process.

The present study established an adverse stress model in mice and cells to evaluate whether curcumin had an inhibitory effect on the proliferation and invasion of glioma induced by adverse psychological stress. In addition, the effect of curcumin on the NE‐induced MAPK signalling pathway and MMP‐2/9 activation was investigated to reveal the antitumour mechanism of curcumin, with the aim of relieving stress.

## 2 MATERIALS AND METHODS

### 2.1 Cell culture and reagents

The human glioma LN229 cell line was obtained from Leibniz Institute DSMZ—German Collection of Microorganisms and Cell Cultures GmbH. The human glioma U87 MG cell line was obtained from American Type Culture Collection. The cells were cultured in DMEM containing 10% foetal bovine serum (FBS, Gibco BRL), 100 U/ml penicillin and 100 mg/ml streptomycin in a humidified atmosphere of 5% CO_2_ at 37°C. Curcumin, propranolol, NE and U0126 were purchased from Sigma‐Aldrich (Merck KGaA).

### 2.2 Nude mouse model of adverse stress restraint

In total, 20 female 4‐6 weeks‐old BALB/c‐nu/nu nude mice were provided by the Institute of Laboratory Animals, Chinese Academy of Medical Sciences [licence no. SCXK (Beijing) 2014‐0013]. Nude mice were housed in a specific‐pathogen‐free environment with fixed room temperature (22 ± 1°C) and humidity (50 ± 5%). The animal experiments were performed in strict accordance with the recommendations of the Guide for the Care and Use of Laboratory Animals published. The protocol was approved by the Committee on the Ethics of Animal Experiments of Weifang Medical University, Shandong, China (approval no. 2019SDL092). Logarithmic growth phase LN229 cells (5 × 10^7 ^cells/ml) were treated with 0.25% trypsin (Gibco; Thermo Fisher Scientific, Inc), suspended in 200 μl serum‐free DMEM and injected subcutaneously into the right axilla of nude mice. One week later, tumour‐bearing mice were randomly divided into four groups: control (DMEM injected), stress (DMEM injected and stressed), curcumin (curcumin injected) and curcumin and stress (curcumin injected and stressed). Five mice were assigned to each group. The nude mice were placed in a 50 ml multi‐well centrifuge tube without squeezing the animal's body during the process in order to establish an adverse stress model through restraint.^4^, ^24^ The restraint stress lasted for 8 hours per day. During rest, the mice were provided free access to food and water. Consistent with previously reported literature and our preliminary results, a single dose of curcumin (60 mg/kg) was administered via an intraperitoneal injection once every other day.^25^ In the control group, curcumin was replaced by DMEM. All groups were maintained for 4 weeks, and all tumour sizes were limited in the range of ethics requirement. Tumour length (A) and width (B) were measured with a calliper every day, and the tumour volume (tumour volume = A × B^2^ × 0.5) was subsequently calculated. At the end of the experiment, the mice in each group were killed with carbon dioxide, and the tumours were excised and stored at −80°C for further analysis.

### 2.3 Analysis of serum NE and EPI levels

Whole blood was extracted from the posterior venous plexus of the eye socket of the mice and was placed at 4℃ for 12 hours and then centrifuged at 356 ×*g* for 20 minutes to obtain serum. EPI and NE concentrations in the serum were measured with an ELISA kit (Rapid Bio Lab, DRE‐H80507C) according to the manufacturer's instructions. Each sample was analysed in triplicate.

### 2.4 Cell proliferation assay

Cell growth was detected using Cell Counting Kit‐8 (CCK‐8, Dojindo Molecular Technologies, Inc) according to the manufacturer‘s instructions. A total of 5 × 10^3^ cells per well were inoculated into 96‐well plates and cultured for 24 hours and then cultured for 24‐72 hours in the presence or absence of curcumin (8, 16, 24 and 32 μmol/L). Next, 10 μl CCK‐8 reagent per well was added and incubated for 2 hours, and then, the absorbance value was measured at 450 nm. The control group was exposed only to DMEM and CCK‐8 reagent. All experiments were repeated three times. The proliferation inhibition rate was calculated as 1–(OD_sample_/OD_control_) × 100%.

### 2.5 Colony formation assay

A total of 100 LN229 and U87 MG cells per well were inoculated into a 6‐well plate and cultured for 24 hours. Next, the cells were treated with or without curcumin (8, 16 and 24 μmol/L) or 10 μmol/L NE. After continuous culture for 12 days, the cells were washed twice with PBS, fixed with 4% paraformaldehyde for 20 minutes and stained with 0.1% crystal violet for 10 minutes. Colonies that contained ≥50 cells were counted under the microscope (magnification, × 100).

### 2.6 Flow cytometry analysis of cell cycle and apoptosis

Glioma cells (1 × 10^6^) were gathered, centrifuged and washed twice with cold PBS. Cells were fixed overnight in 70% ethanol, then stained with propidium iodide (Cell Cycle Detection kit; Beyotime, C1052) and analysed with flow cytometer (BD Accuri C6 Plus; BD FACSAria ll). To detect apoptosis, cells were stained with Annexin V‐FITC and propidium iodide (Apoptosis Detection kit; BD, 556 547) as recommended by the manufacturer and analysed by flow cytometry.

### 2.7 Wound healing assay

LN229 and U87 MG cells were seeded in 6‐well plates at 5 × 10^5^ cells per well, and cells were incubated for 24 hours until reaching ~90% confluence. A linear wound was created by scraping with a 10 µl pipette tip on a monolayer of cells. Cells were pre‐treated with or without curcumin (8, 16 and 24 μmol/L) for 4 hours and then treated with or without NE (10 μmol/L) for 24 hours. Wound healing was observed using a microscope (magnification, × 100) and photographed.

### 2.8 Transwell Matrigel invasion assay

After pre‐treatment of LN229 and U87 MG cells without or with curcumin (8, 16 and 24 μmol/L) for 4 hours, the cells (1.5 × 10^5^) were suspended in 200 μl DMEM (with or without 10 μmol/L NE) and placed in the upper chamber of Transwell polycarbonate membrane (pore size, 8 μmol/L) chamber (Corning Inc) with Matrigel and 600 μl DMEM containing 10% foetal calf serum was placed in the lower chamber. After 24 hours of culture, the unperforated cells on the surface of the membrane were removed with a cotton swab. Cells were fixed with 4% paraformaldehyde for 15 minutes at room temperature. The membrane was stained with 0.1% crystal violet and photographed (magnification, × 200). The number of transmembrane cells was counted in five randomly selected fields of view, and the results are expressed as the mean ± standard deviation (SD).

### 2.9 Gelatin zymography analysis

The activity of MMP‐2 and MMP‐9 secreted by LN229 cells was determined by gelatin zymography. LN229 cells were seeded in 6‐well plates, allowed to grow to ~90% confluence for 24 hours and then maintained in serum‐free medium. The cells were pre‐treated with or without curcumin (24 μmol/L) for 4 hours and then treated with or without NE (10 μmol/L) for 24 hours. Subsequently, the conditioned medium was collected and subjected to 10% SDS‐PAGE in the presence of 0.1% gelatin. Samples were not boiled prior to electrophoresis. After electrophoresis, the gels were washed three times for 15 minutes each at room temperature in an eluate with or without 2.5% Triton X‐100 to remove SDS. Next, the gels were incubated in a solution containing 50 mmol/l Tris‐HCL, 5 mmol/l CaCl_2_, 1 μmol/l ZnCl_2_ and 0.02% Brij‐35 for 42 hours at 37°C. The gels were subsequently stained with 0.25% Coomassie Brilliant Blue (G250) in 10% acetic acid (v/v) and 30% methanol (v/v) and then de‐stained with the same solution but without Coomassie Brilliant Blue. Gelatinolytic activity was detected as unstained bands on a blue background.

### 2.10 Reverse transcription‐quantitative PCR (RT‐qPCR) analysis

Total RNA was extracted using TRIzol^®^ (Invitrogen; Thermo Fisher Scientific, Inc) according to the manufacturer's instructions, and then, 1 μg total RNA was used to synthesize cDNA using PrimeScript™ RT Master Mix (Takara Biotechnology Co., Ltd.). Using cDNA as a template, amplification was performed for 35 cycles using the SYBR^®^ Premix Ex Taq^TM^ II kit (Takara Biotechnology Co., Ltd.), and gene‐specific primer sequences were designed based on the literature.^26^ The primer sequences are as follows: MMP‐2 5'‐CAAGGACCGGTTTATTTGGC‐3' (forward) and 5'‐ATTCCCTGCGAAGAACACAGC‐3' (reverse); MMP‐9 5'‐TTGACAGCGACAAGAAGTGG‐3' (forward) and 5'‐GCCATTCACG ‐TCGTCCTTAT‐3' (reverse); and GAPDH 5'‐GCACCGTCAAGGCTGA‐GAAC‐3' (forward) and 5'‐TGGTGAAGACGCCAGTGGA‐3' (reverse). The primers were synthesized by Bao Bio Biotechnology Co., Ltd. The relative gene expression was calculated using the 2^‐ΔΔCq^ method as previously described. ^27^

### 2.11 Western blotting

Protein expression levels were determined using standard Western blot methods. Protein samples were extracted from glioma tissues or cells, and the protein concentration was determined with the BCA Protein Assay Kit (Beyotime Institute of Biotechnology, cat. no. P0012). Next, protein samples were separated by SDS‐PAGE and then transferred to nitrocellulose membranes, and 5% skimmed milk powder was used for blocking for 1 hours, followed by incubation with the following antibodies: anti‐MMP‐2, anti‐MMP‐9, Bcl‐2 and Bcl‐XL (all 1:1,000; Abcam; cat. nos. ab86607, ab236494, ab32124 and ab32370); anti‐ERK1/2 and anti‐p‐ERK1/2 (both 1:5,000; CST, Inc cat. nos. 4695 and 8544); anti‐p38, anti‐p‐p38, anti‐JNK, anti‐p‐JNK, CDK4 and CDK6 (all 1:1,000; CST, Inc); and anti‐CD147 (1:2,000; R&D Systems Inc; cat. No. MAB972‐100). Antibody incubations were carried out at 4°C overnight, followed by three washes for 10 minutes each with TBS containing Tween‐20, and incubated with the secondary antibodies at room temperature for 1.5 hours. Subsequently, the signal was visualized with enhanced chemiluminescence reagent, following the manufacturer's protocol (Thermo Fisher Scientific, Inc; cat. no. 32 209).

### 2.12 Construction of shRNA targeting CD147, and transfection of LN229 and U87 MG cells

The construction of the interference sequence for down‐regulating the CD147 gene was completed by Shanghai GeneChem Co., Ltd. According to the cDNA sequence of GenBank CD147 (NM_001728) and a previous study,^28^ the effective interference sequence was shRNA‐CD147 (808‐828 bp), 5'‐TGACAAAGGCAAGAACGTC‐3', and the designed negative control sequence was 5'‐ACTACCGTTGTTATAGGTG‐3'. The restriction sites for *Age*I and *Eco*RI were, respectively, added to both ends of these oligonucleotide sequences, according to vector requirements. Two complementary single strands of DNA were synthesized, annealed to form a double strand and ligated with the linearized vector GV248 using T_4_ DNA ligase. After the recombinants were correctly sequenced, the virus was packaged using 293T cells. LN229 and U87 MG cells were seeded in 6‐well plates (1 × 10^5 ^cells/well). When the cell density reached 30% after 24 hours, the negative and shRNA‐CD147 lentiviral fluid was used to infect the cells with a multiplicity of infection of 30. When the fusion rate was ~80%, the protein expression of CD147, MMP‐2 and MMP‐9 was measured.

### 2.13 Statistical analysis

Statistical analysis was performed using GraphPad Prism 5 software (GraphPad Software, Inc), and all data are expressed as the mean ± SD. Differences between two groups were evaluated by Student's *t* test, and differences among groups were assessed by one‐way analysis of variance (ANOVA). *P* < .05 was considered to indicate a statistically significant difference.

## 3 RESULTS

### 3.1 Curcumin inhibits the psychological stress‐induced growth of transplanted tumours in nude mice

**FIGURE 1:**
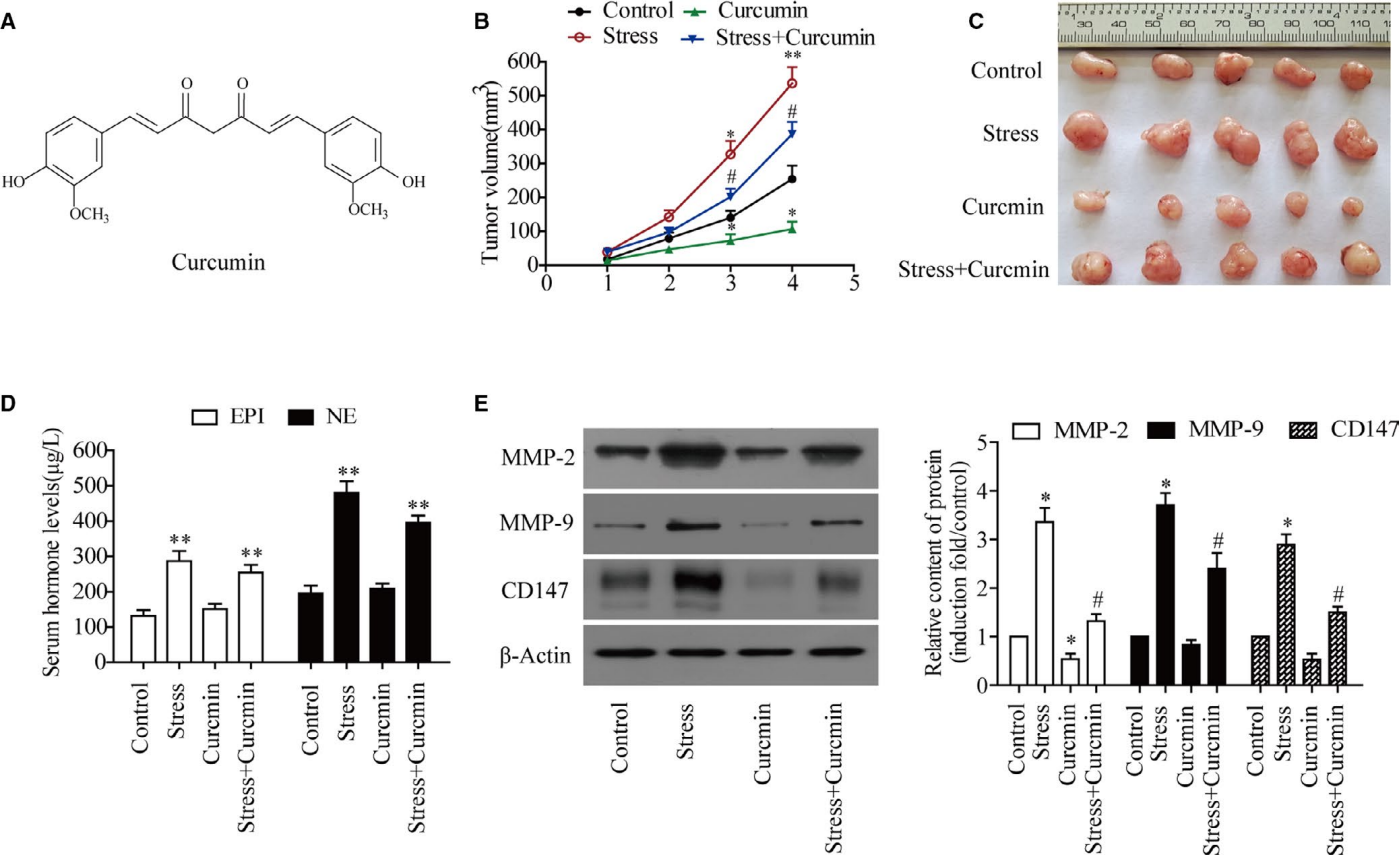
Curcumin inhibited the growth and protein expression of transplanted tumours in nude mice with adverse psychological stress. A, Chemical structure of curcumin. B, Effect of curcumin on the growth of transplanted tumours in nude mice (n = 5). C, Tumour images of nude mice in each group. D, ELISA was used to detect the levels of epinephrine and norepinephrine in the serum of nude mice. E, Western blotting was used to detect the effect of curcumin on the expression of MMP‐2, MMP‐9 and CD147 protein in tumour tissues of nude mice. ^*^ *P* < .05, ^**^ *P* < .01 vs. control group; ^#^ *P* < .05 vs. stress group

Previous reports have demonstrated that curcumin (Figure 1A) inhibited the growth of xenografted tumours in nude mice. ^25^ To evaluate the effect of curcumin on tumour proliferation induced by adverse psychological stress, the progression of post‐implantation tumour growth was examined. The results showed that the tumour volume of the control group was smaller than that of the stress group from the third week to the end of the experiment.

Moreover, curcumin was used to treat stressed nude mice. It was found that the tumour volume of the stress and curcumin group was significantly smaller than that of the stress only group. These results suggested that curcumin was able to inhibit tumour growth induced by psychological stress (Figure 1B‐C). To evaluate whether each group of nude mice was in a state of stress, blood samples were collected to determine the levels of serum stress hormones in each group. The results showed that the levels of EPI and NE in the stress group were significantly higher than those in the non‐stress group. Remarkably, serum NE was elevated by 2.5‐fold in nude mice with psychological stress (Figure 1D). However, there was no impact on NE or EPI levels with curcumin treatment. In addition, Western blot analysis showed that psychological stress could induce the expression of MMP‐2, MMP‐9 and CD147, which was inhibited by curcumin (Figure 1E).

### 3.2 Curcumin inhibited NE‐induced proliferation of glioma cells

**FIGURE 2:**
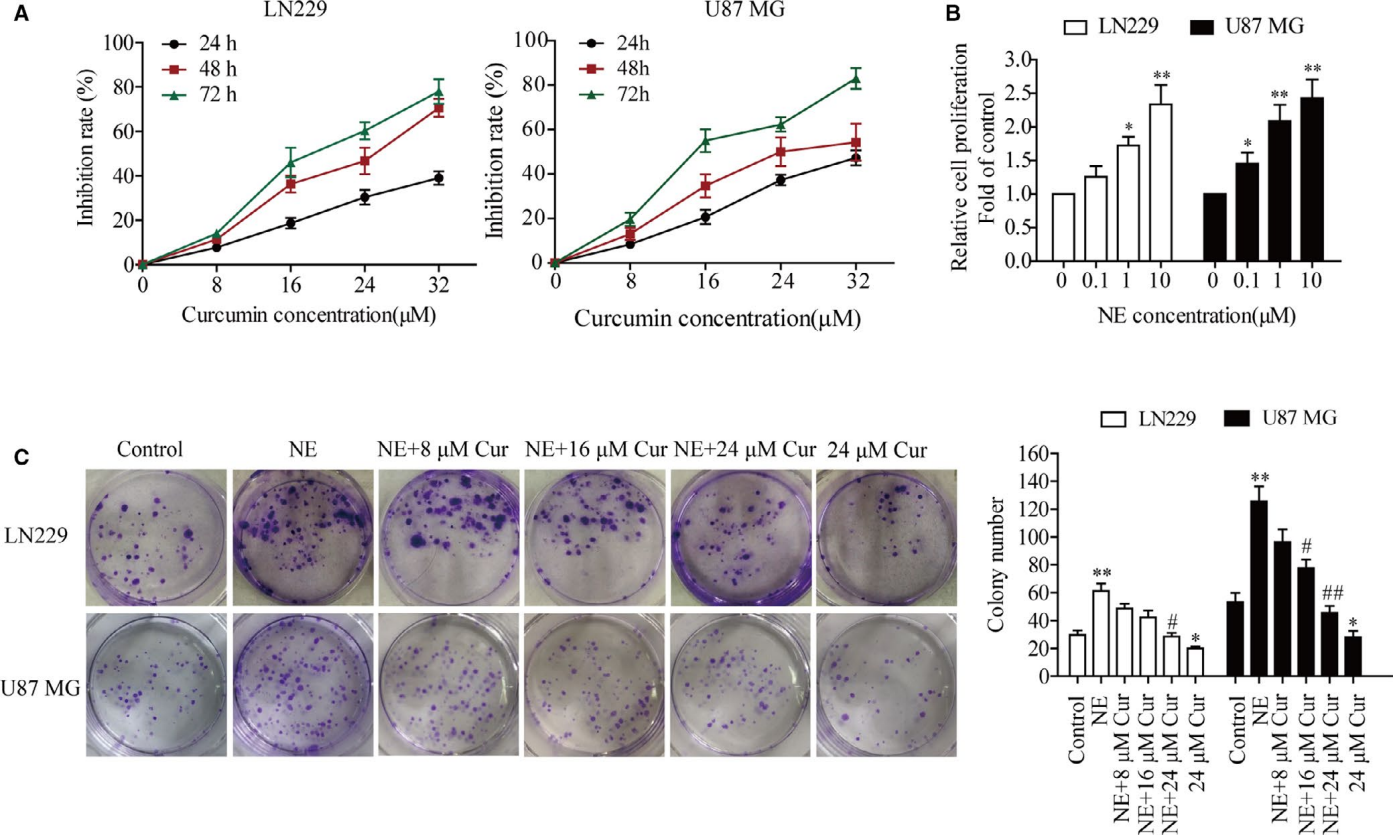
Curcumin dose‐dependently inhibited the NE‐induced proliferation of glioma cells. A, Glioma LN229 and U87 MG cells were treated with different concentrations of curcumin (0, 8, 16, 24 or 32 μmol/L) for 24, 48 or 72 h, and the inhibition ratio of cancer cell proliferation was analysed with the CCK‐8 assay. B, LN229 and U87 MG cells were cultured with various concentrations of NE (0, 0.1, 1 or 10 μmol/L) for 48 h, and then, cell proliferation was assessed with the CCK‐8 assay. C, LN229 and U87 MG cells were treated with or without curcumin (8, 16 or 24 μmol/L) or 10 μmol/L NE to evaluate colony forming ability. ^*^ *P* < .05, ^**^ *P* < .01 vs. control group; ^#^ *P* < .05 vs. NE group

Previous studies have shown that curcumin elicits an antiproliferative activity against numerous tumour types. ^19^, ^20^ To investigate the killing effect and cytotoxicity of curcumin on glioma cells, LN229 and U87 MG cells were treated with different concentrations of curcumin (8, 16, 24 or 32 μmol/L) for 24, 48 and 72 hours. The proliferative capacity of the cells decreased in time‐dependent and dose‐dependent manner. Curcumin showed a half maximal inhibitory concentration of 24 μmol/L and had no cytotoxic effect in LN229 or U87 MG cells (Figure 2A). Therefore, 8, 16 and 24 μmol/L were the treatment concentrations of curcumin used in subsequent experiments.

Exogenous NE was used to stimulate glioma LN229 and U87 MG cells to simulate the stress environment in vitro, ^29^ and it was found that NE enhanced the proliferation of LN229 and U87 MG cells in a dose‐dependent manner, and the maximal stimulatory effect was observed at a dose of 10 μmol/L (Figure 2B). In order to further study the effect of curcumin on NE‐induced proliferation of LN229 and U87 MG cells, the cells were exposed to 10 μmol/L NE without or with curcumin (8, 16 or 24 μmol/L). These results showed that the colony formation ability of LN229 and U87 MG cells was increased after NE treatment, while curcumin could inhibit the NE‐induced colony formation of LN229 and U87 MG cells in a dose‐dependent manner (Figure 2C).

### 3.3 Curcumin inhibited NE‐triggered G1 to S phase transition and apoptosis in glioma cells

**FIGURE 3:**
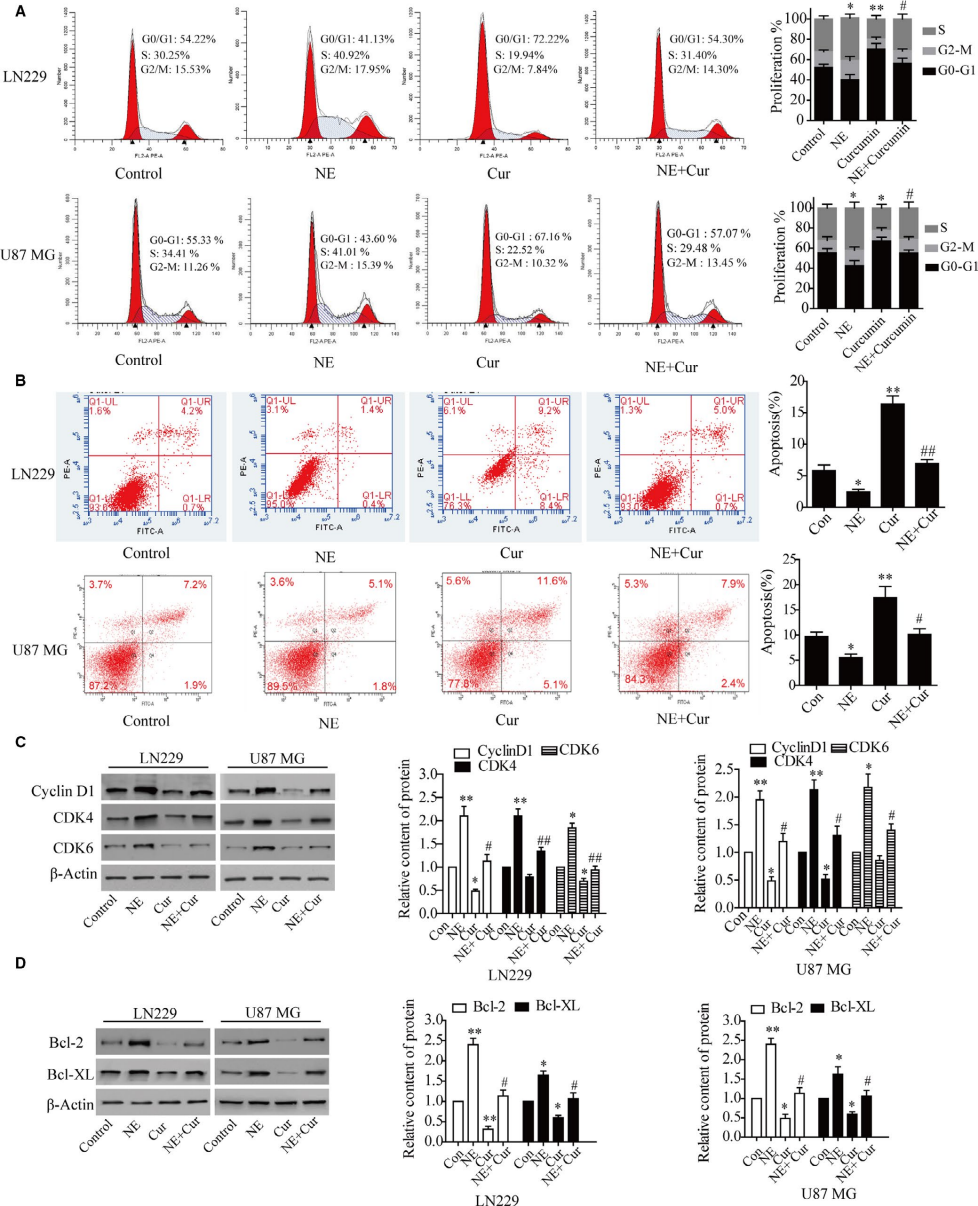
The effect of curcumin on cell cycle distribution and apoptosis of glioma cells treated with NE. A, Glioma cells were pre‐treated with or without curcumin (24 μmol/L) for 4 h, followed by exposure to 10 μmol/L NE for 24 h except those in control group, and cell cycle was determined by flow cytometry. B, Apoptosis was detected by flow cytometry using Annexin V‐FITC/ PI kit. C, D, The expression of cyclin D1, CDK4/6, Bcl‐2 and Bcl‐XL was detected by Western blotting. ^*^P < .05, ^**^P < *.01 vs*. control group; ^#^ *P* < .05, ^##^ *P* < *.01* vs. NE group

To investigate the mechanism of curcumin inhibiting the NE‐induced cell proliferation, cell cycle and apoptosis were analysed by flow cytometry. Then, Western blot analysis was performed to investigate the effect of curcumin on cyclin D1, CDK4/6, Bcl‐2 and Bcl‐XL in glioma cells. The results demonstrated that curcumin inhibited NE‐triggered G1 to S phase transition in glioma cells and reduced expression of the cyclin D1 and CDK4/6 (Figure 3A‐C). Curcumin could also induce apoptosis of glioma cells treated with NE and reduce the expression of anti‐apoptotic proteins Bcl‐2 and Bcl‐XL (Figure 3B‐D).

### 3.4 Curcumin inhibits the NE‐induced migration and invasion of glioma cells

**FIGURE 4:**
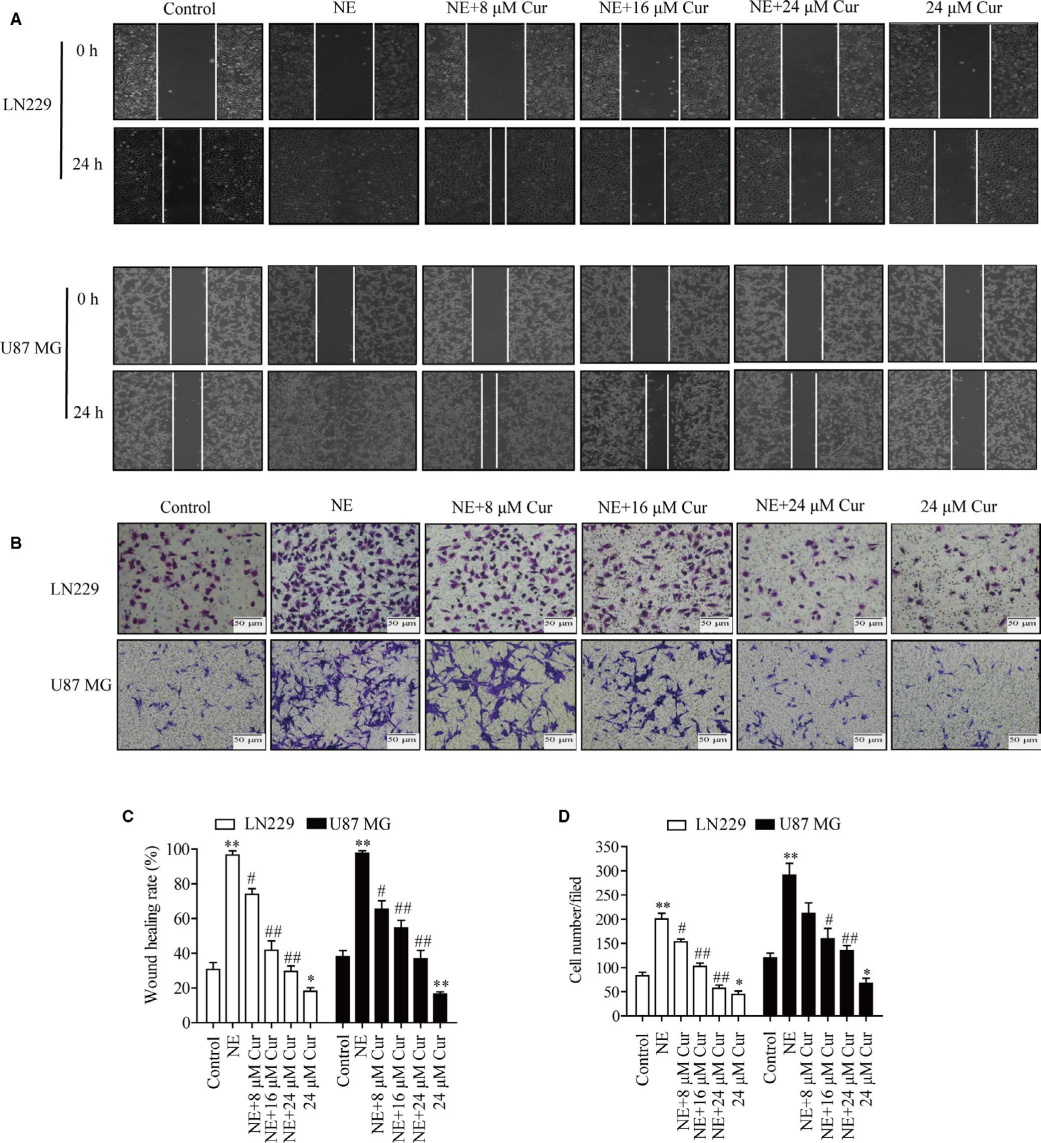
Curcumin inhibited NE‐induced migration and invasion of glioma cells in a dose‐dependent manner. A, LN229 and U87 MG cells were treated with curcumin (8, 16 or24 μmol/L) for 4 h followed by treatment with NE (10 μmol/L) to evaluate cell migration ability using a wound healing assay (magnification, × 100). B, After subjecting the cells to the same treatment as described in panel A, the cell invasive ability was evaluated by Transwell assay (magnification, × 200). C, D, Statistical histograms of wound healing and Transwell assays. ^*^ *P* < .05, ^**^ *P* < .01 vs. control group; ^#^ *P* < .05, ^##^ *P* < .01 vs. NE group

Glioma LN229 and U87 MG cells were exposed to NE without or with curcumin (8, 16 or 24 μmol/L), and changes in migration and invasion abilities of glioma cells were observed. The results showed that curcumin‐treated cells exhibited a dose‐dependent delay during wound healing (Figure 4A‐C). NE exposure significantly enhanced the invasive ability of LN229 and U87 MG cells, but as the curcumin concentration increased from 8 to 24 μmol/L, the number of cells invading the inferior cavity decreased (Figure 4B‐D). These results suggest that curcumin may be a potent inhibitor of NE‐induced glioma cell migration and invasion.

### 3.5 Curcumin down‐regulated the NE‐induced secretion and expression of MMPs in glioma cells

**FIGURE 5:**
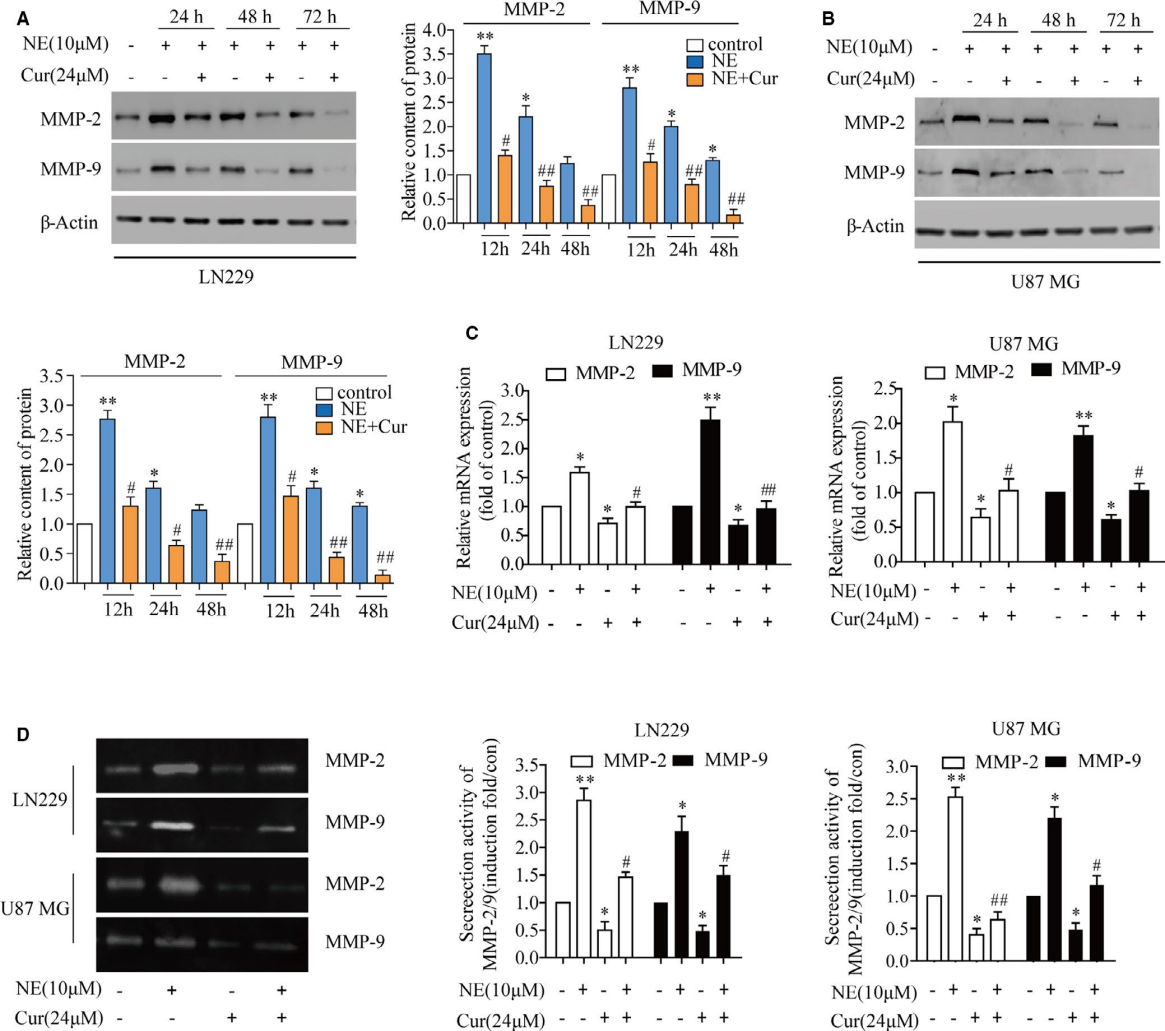
Curcumin decreased the secretion and expression of MMP‐2 and MMP‐9 induced by NE in glioma cells. A, B, Cells were pre‐treated with or without 24 μmol/L curcumin for 4 h and then treated with or without 10 μmol/L NE for 24, 48 and 72 h, respectively. The protein expression of MMP‐2 and MMP‐9 was detected by Western blotting. C, Cells were pre‐treated with curcumin (24 μmol/L) for 4 h and then treated with NE (10 μmol/L) for 24 h. And then, reverse transcription‐quantitative PCR was used to detect the expression of MMP‐2 and MMP‐9 at the RNA level. D, Cells were pre‐treated with curcumin (24 μmol/L) for 4 h and then treated with NE (10 μmol/L) for 24 h. The conditioned medium was collected, and the gelatinase activity was detected. ^*^ *P* < .05, ^**^ *P* < .01 vs. control group; ^#^ *P* < .05, ^##^ *P* < .01 vs. NE group

To determine the effect of curcumin on NE‐induced MMP‐2 and MMP‐9 expression, Western blotting, RT‐qPCR and zymography were performed in LN229 and U87 MG cells. Western blot analysis showed that NE significantly increased the expression of MMP‐2 and MMP‐9 for 24, 48 and 72 hours. NE‐induced protein expression levels were evidently suppressed by curcumin in a time‐dependent manner (Figure 5A,B). RT‐qPCR demonstrated that NE could induce the expression of MMP‐2 and MMP‐9 in LN229 and U87 MG cells at the RNA level and that curcumin blocked the up‐regulation of MMP‐2 and MMP‐9 induced by NE (Figure 5C). Zymogram analysis revealed that NE treatment increased the secretion of MMP‐2 and MMP‐9 in LN229 cells and that curcumin could inhibit these NE‐mediated effects (Figure 5D). These results indicated that curcumin inhibited NE‐induced invasion in glioma, which was associated with the down‐regulation of the expression and secretion of MMP‐2 and MMP‐9.

### 3.6 Curcumin down‐regulated NE‐induced expression of MMP‐2 and MMP‐9 by blocking the ERK/MAPK signalling pathway

**FIGURE 6:**
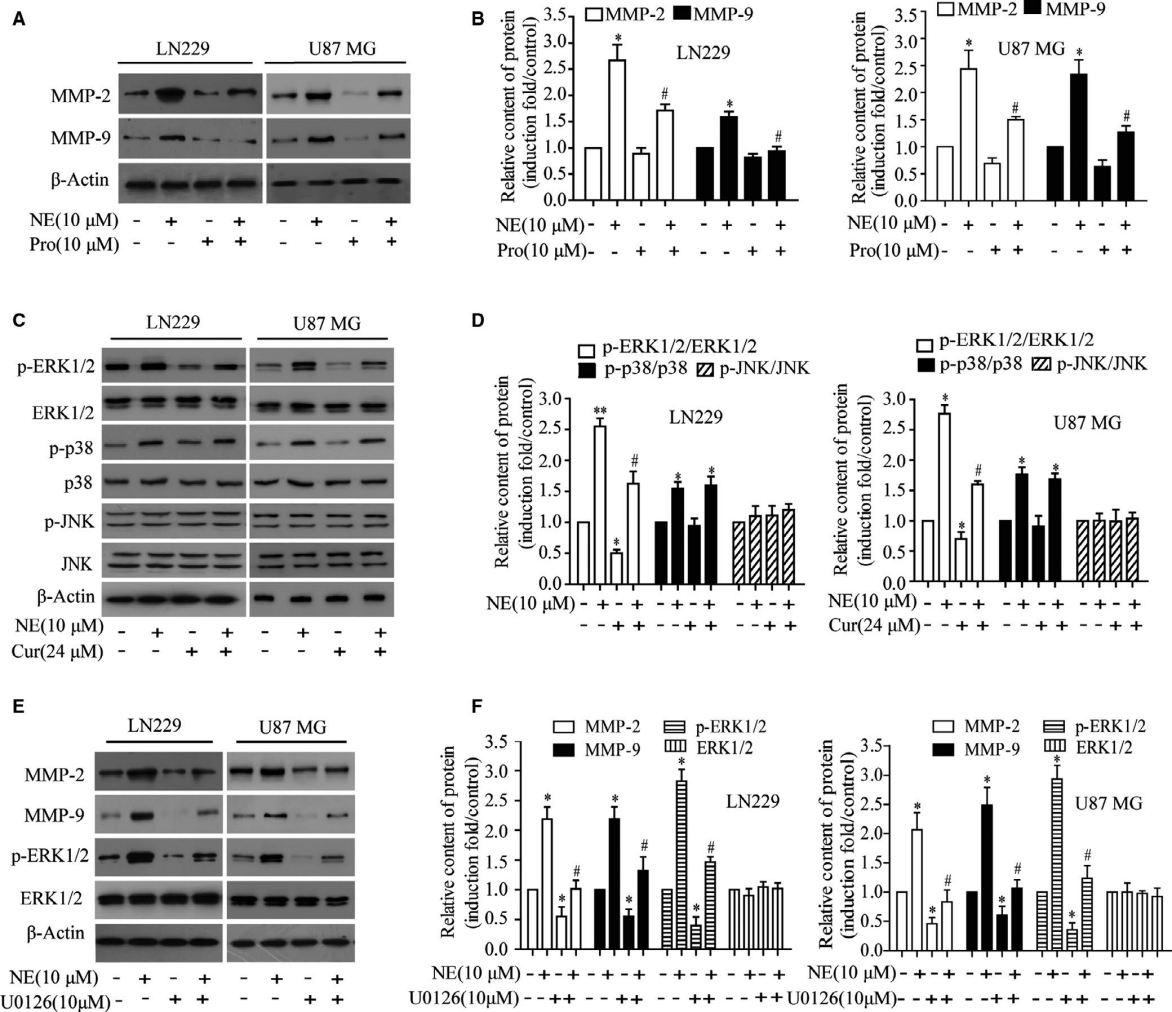
Effect of curcumin on NE‐induced MAPK activities, and its correlation with MMP‐2 and MMP‐9 regulation. A,B Cells were incubated with propranolol (10 μmol/L) for 30 min and then with NE (10 μmol/L) for 24 h. Next, the expression of MMP‐2 and MMP‐9 was determined. C, D, Cells were pre‐treated with curcumin (24 μmol/L) for 4 h and then treated with NE (10 μmol/L) for 30 min. Phosphorylated and total MAPK (ERK1/2, p38 and JNK) was detected by Western blotting. E, F, Treatment of LN229 cells with the ERK1/2 inhibitor U0126 (10 μmol/L) for 30 min, followed by NE (10 μmol/L) induction for 24 h. And then, the expression of MMP‐2 and MMP‐9 was detected by Western blotting. ^*^ *P* < .05 vs. control group; ^#^ *P* < .05 vs. NE group

To investigate the mechanism of curcumin inhibiting the expression of MMP‐2 and MMP‐9 induced by NE, LN229 and U87 MG cells were pre‐treated with the β‐blocker propranolol, and it was found that propranolol could inhibit NE‐induced MMP‐2 and MMP‐9 expression (Figure 6A,B). These results suggested that the increased expression of MMP‐2 and MMP‐9 was induced by NE through the β‐AR receptor pathway. NE was shown to stimulate MAPK signalling pathways through the β‐AR receptor in a variety of tumours, which is involved in tumour migration and invasion. Phosphorylated or total MAPK (p38 MAPK, ERK1/2 and JNK) levels was analysed by Western blotting. The results showed that NE could induce the phosphorylation of ERK1/2 and p38 MAPK. However, curcumin only inhibited the phosphorylation of ERK1/2 (Figure 6C,D). Subsequently, ERK1/2 expression was blocked with U0126, which resulted in reduced MMP‐2 and MMP‐9 expression (Figure 6E,F). These results suggested that NE regulated MMP‐2 and MMP‐9 expression through the β‐AR‐ERK/p38 pathway, while the specific inhibition of ERK1/2 phosphorylation by curcumin was directly involved in the regulation of NE‐induced MMP‐2 and MMP‐9 expression.

### 3.7 Decreased CD147 expression inhibited NE‐induced expression of MMP‐2 and MMP‐9 in glioma cells

**FIGURE 7:**
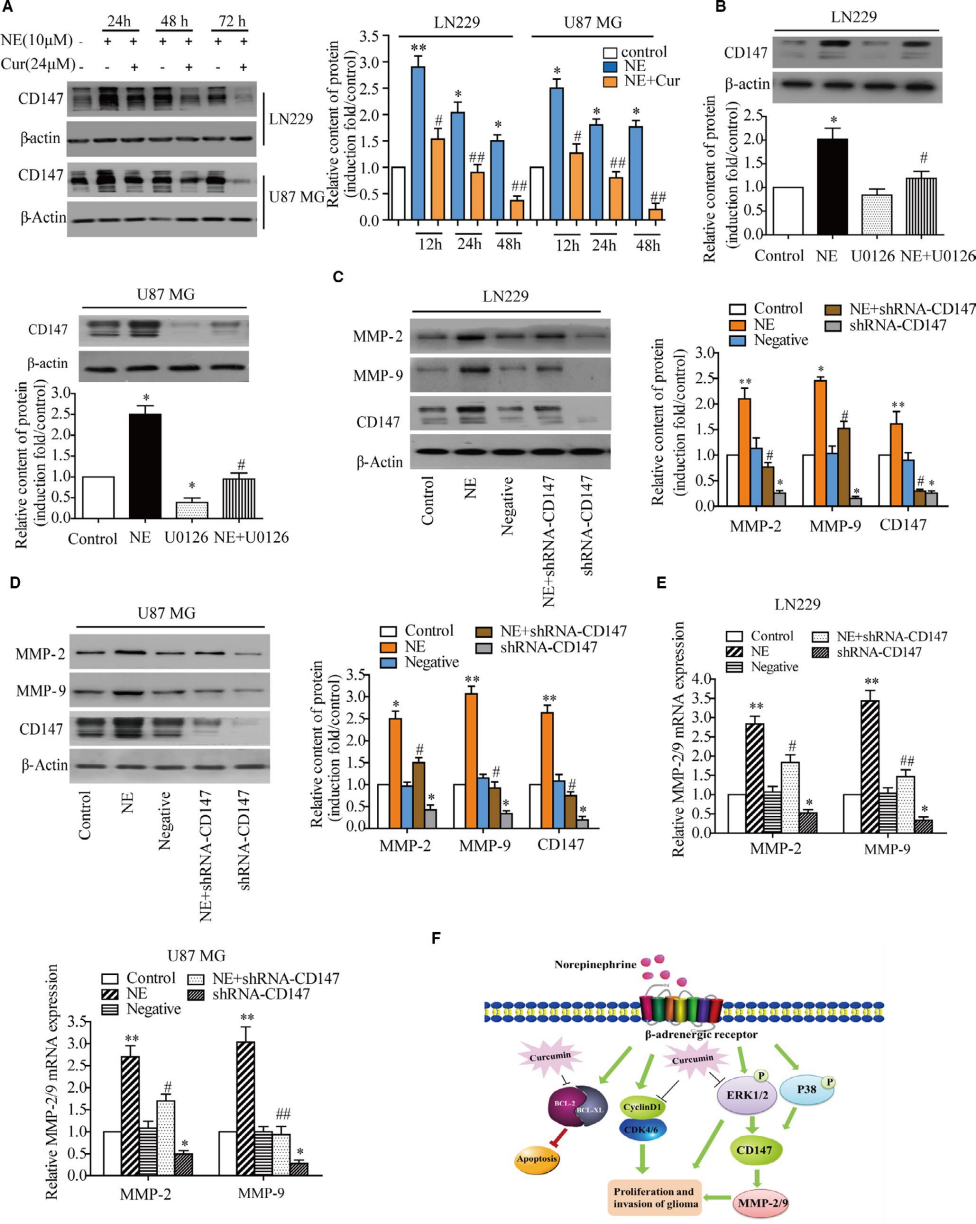
Decreased CD147 expression inhibits NE‐induced MMP‐2 and MMP‐9 expression. A, Cells were pre‐treated with or without 24 μmol/L curcumin for 4 h and then treated with or without 10 μmol/L NE for 24, 48 and 72 h. The protein expression of CD147 was detected by Western blotting. B, Cells were pre‐treated with U0126 (10 μmol/L) for 30 min and then treated with NE (10 μmol/L) for 24 h. Next, Western blotting was used to detect the expression of CD147. C,D shRNA‐CD147 and its negative control were transfected into LN229 and U87 MG cells, which were then treated with NE (10 μmol/L) for 24 h. The expression of MMP‐2 and MMP‐9 was detected by Western blotting. E, MMP‐2 and MMP‐9 mRNA expression was detected by reverse transcription‐quantitative PCR. F, Schematic illustration of the underlying mechanism of curcumin inhibited glioma proliferation and invasion with NE stimulation. ^*^ *P* < .05, ^**^ *P* < .01 vs. control group; ^#^ *P* < .05, ^##^ *P* < .01 vs. NE group

CD147 is a transmembrane glycoprotein that induces MMPs and participates in carcinoma invasion. Western blot assays were performed to investigate whether the expression of CD147 was down‐regulated by curcumin. As shown in Figure 7A, NE significantly increased the expression of CD147 for 24, 48 and 72 hours. Pre‐treatment with curcumin strongly inhibited the NE‐induced expression of CD147 at the protein level. It has been demonstrated that the expression of CD147 is positively controlled by the MAPK (p38, ERK and JNK) signalling pathway.^30^, ^31^ In order to determine the role of ERK signalling molecules in NE‐induced CD147 and its downstream MMP‐2 and MMP‐9, U0126 was used to pre‐treat LN229 and U87 MG cells, and it was found to be able to block NE‐induced CD147 expression (Figure 7B). Subsequently, it was observed that the expression of NE‐induced MMP‐2 and MMP‐9 was also decreased by reduced CD147 expression via RNA interference (Figure 7C‐E). Taken together, the results suggest that NE increases the expression of CD147 by activating the ERK signalling pathway in glioma cells and then induces the expression of MMP‐2 and MMP‐9 to promote tumour invasion. Besides, NE promotes the proliferation of glioma cells by regulating the expression of cyclin D1/CDK4/6 and Bcl‐2/Bcl‐XL, while curcumin can reverse these effects to alleviate tumour progression induced by adverse psychological stress (Figure 7F).

## 4 DISCUSSION

As a natural pharmaceutical compound, curcumin can penetrate the blood‐brain barrier, so it has a good therapeutic effect on primary central nervous system tumours including GBM. ^32^ Curcumin has been shown to play an anti‐GBM role through regulation of proliferation, apoptosis, metastasis, invasion, autophagy and potential molecular targets, including Wnt/β‐catenin, JAK/STAT3, NF‐κB and MAPK. ^33^, ^34^ However, few studies have investigated whether curcumin could inhibit stress‐induced tumour invasion, and the related mechanism is unknown.

Previous animal experiments have revealed that psychosocial factors, especially adverse stress, can accelerate the growth and metastasis of tumours by inducing the release of catecholamine neurotransmitters.^35^, ^36^ The present results showed that the serum levels of EPI and NE were significantly increased in glioma‐bearing nude mice with adverse stress, and the growth of the transplanted tumour was accelerated. Experiments at the cellular level further confirmed these findings, and stress hormone NE promoted the proliferation, migration and invasion of glioma LN229 and U87 MG cells. It was also found that curcumin could significantly reverse these phenomena, and its mechanism was closely associated with the inhibition of the MAPK/ERK signalling pathway.

MMPs are important molecules that promote tumour growth and invasion. Aberrant expression of MMPs leads to a variety of pathological conditions, including tumour cell proliferation and invasion. Recent studies have shown that psychological stress can improve the activity of MMPs in mice. Wu found that MMP‐2, MMP‐9 and matrix‐type MMP‐1 mRNA expression increased in colon tumours and liver tissues of socially isolated stressed mice compared with that of the control group. ^37^ The present study revealed similar effect in glioma cells, that is, that adverse stress promoted the expression and secretion of MMP‐2 and MMP‐9, while this phenomenon was inhibited by curcumin. The role of MAPK in the activation of MMPs expression is well known as a downstream modulator of NE. Recent studies have shown that NE can stimulate pancreatic cancer proliferation, migration and invasion through β‐AR‐dependent activation of the P38 MAPK pathway. ^22^ In SH‐SY5Y neuroblastoma cells, NE treatment activates the ERK and JNK pathways and increases the transcriptional activity of activator protein‐1(AP‐1), which is associated with neuronal plasticity. ^23^ The results of the present study demonstrate that NE could activate the ERK and P38 MAPK pathways in glioma LN229 and U87MG cells. These studies suggest that the MAPK signalling pathway activated by NE may be depending on the cell type.

In recent years, the effect of curcumin on MAPK signalling pathway has been widely concerned. Woo et al found that curcumin inhibits the expression of MMP‐9 as well as the growth and invasion of glioma induced by phorbol myristate acetate by inhibiting the phosphorylation of ERK1/2, JNK and p38 MAPK. ^38^ Liang et al also found that curcumin effectively inhibited EMT induced by tobacco smoke through suppressing ERK and JNK pathways, but did not affect the level of p38 MAPK in gastric cancer cells. ^39^ In the present study, curcumin down‐regulated the expression of MMP‐2 and MMP‐9 induced by NE in gliomas by blocking the ERK signalling pathway, while p38 MAPK pathway was not actively involved in this process. Overall, the effect of curcumin on MAPK pathway is inconsistent in different studies. It may be due to different cell lines and culture conditions, which needs to be further determined.

The ERK/MAPK signalling pathway is a key regulator of cell proliferation and survival. Previous studies have shown that ERK pathway is required for the progression of cells from the G0/G1 to S phase.^40^ Activation of the ERK pathway could induce the expression of cell cycle regulators cyclin D1 and Bcl‐2. ^41^, ^42^ We hypothesized that curcumin may inhibit the ERK pathway, which lead to cell cycle arrest and increase apoptosis, and thus inhibit the proliferation of glioma induced by adverse psychological stress, but more studies are required to further elucidate this.

Abnormal activation of the ERK signalling pathway may trigger the CD147 signalling pathway in tumour cells. ^43^, ^44^ CD147 is highly expressed in a variety of tumours and, when activated by certain molecules, induces the production of downstream MMP‐2 and MMP‐9, and is involved in cell migration and invasion.^45^ The findings of the present study also confirmed that NE induced CD147 expression by activating ERK1/2 in LN229 and U87 MG cells. In addition, NE‐induced MMP‐2 and MMP‐9 up‐regulation can be restored by siRNA‐CD147. These results suggest that CD147 is involved in the regulation of the ERK1/2/ MMP‐2 /MMP‐9 signalling pathway by NE. However, the mechanism by which NE activates ERK1/2 and CD147/MMP‐2/MMP‐9 needs further study.

In conclusion, these data suggest that curcumin effectively alleviated the invasion of glioma promoted by adverse psychological stress through inhibiting the MAPK/ERK signalling pathway which reduced the expression of CD147 and MMP‐2/9. Furthermore, curcumin induced cell cycle changes and increased apoptosis and then inhibited cell proliferation. Therefore, curcumin may be a promising drug for preventing and treating the progression of glioma due to adverse psychological stress.

## CONFLICT OF INTEREST

All authors confirm that there are no conflicts of interest.

## AUTHOR CONTRIBUTION

**Ping Wang:** Data curation (equal); Investigation (equal); Project administration (equal); Writing‐original draft (lead); Writing‐review & editing (equal). **Xinwei Hao:** Data curation (equal); Investigation (equal); Methodology (equal). **Xiaohan Li:** Investigation (equal); Methodology (equal). **Yizhi Yan:** Investigation (supporting); Methodology (supporting); Project administration (equal). **Wenxiu Tian:** Investigation (supporting); Project administration (supporting). **Lin Xiao:** Methodology (supporting); Project administration (supporting). **Zhenming Wang:** Investigation (equal); Software (supporting). **Junhong Dong:** Conceptualization (lead); Data curation (equal); Funding acquisition (lead); Writing‐original draft (supporting); Writing‐review & editing (equal).

## Data Availability

The data that support the findings of this study are available from the corresponding author upon reasonable request.
